# Associations between low parental education, childhood adversities and sickness absence in midlife public sector employees

**DOI:** 10.1177/14034948221087996

**Published:** 2022-05-12

**Authors:** Aino Salonsalmi, Ossi Rahkonen, Eero Lahelma, Olli Pietiläinen, Tea Lallukka

**Affiliations:** Department of Public Health, University of Helsinki, Finland

**Keywords:** Sick leave, life course epidemiology, childhood adversity

## Abstract

**Aims:**

Parental education and childhood adversities are associated with long-term work disability but their contribution to sickness absence is largely unknown. We aimed to examine the associations between parental education, childhood adversities and self-certified and medically-certified sickness absence among midlife employees.

**Methods:**

The Helsinki Health Study baseline survey data (2000–2002) of 40-to-60-year-old municipal employees were linked with sickness absence data from the employer’s register. Self-certified (1–3 days) and medically-certified (>3 days) sickness absence spells were followed from 2003 until the end of 2008. The study included 5728 employees. The analyses were made by Poisson regression and the results are presented as rate ratios (RRs) and their 95% confidence intervals (CIs).

**Results:**

Low maternal education was associated with self-certified sickness absence (RR 1.32, 95% CI 1.13–1.55) among women only whereas both low maternal (1.49, 1.26–1.77) and low paternal education (1.48, 1.32–1.67) were associated with medically-certified sickness absence. Adjustment for own occupational class mainly abolished these associations. Having experienced any childhood adversity was associated with self-certified (1.18, 1.12–1.25) and medically-certified (1.22, 1.15–1.30) sickness absence. In addition, childhood economic difficulties, childhood illness, parental divorce, parental mental illness, parental alcohol problems and bullying were each associated both with self-certified and with medically-certified sickness absence. The associations mainly remained after adjustments for occupational class, marital status, working condition, body mass index and health behaviours.

**Conclusions:**

**Low parental education and childhood adversities contributed to midlife sickness absence. Promoting well-being of families with children might help sustain adult work ability and prevent sickness absence still in midlife.**

## Introduction

There is increasing evidence that childhood socioeconomic circumstances and childhood adversities have long-term influences on adult health [1]. Such influences might be due to biological and social conditions at vulnerable periods having direct long-term effects on health or influencing through adult conditions or due to risk factors accumulating throughout the life-course [2]. Midlife is a key period in the life course because it connects early and late periods of life and balances growth and decline [3]. Childhood factors have been shown to influence adult health still in midlife [3–5]. As ill health is a key domain of work disability, childhood factors might also contribute to work disability. Previous studies have found that low childhood socioeconomic position and childhood adversities are associated with disability retirement [6,7] whereas studies examining short-term work disability such as sickness absence are rare [6,8–10]. The scarcity of research may be partly due to lack of longitudinal studies and reliable register data on sickness absence.

Most previous studies have focused on childhood socioeconomic position. A Norwegian study on young adults found that risk of sickness absence due to musculoskeletal diseases increased with decreasing parental educational level [9]. A Swedish study on young adults found that low paternal socioeconomic position was associated with sickness absence [8]. A UK study examining a birth cohort born in 1958 found that low paternal socioeconomic position was associated with long-term sickness absence in midlife [10].

In addition to childhood socioeconomic position some other childhood adversities have been examined. A Finnish study found that childhood adversity was associated with work disability due to any cause, due to mental disorders and due to musculoskeletal diseases [6]. Each individual adversity such as divorce/separation of the parents, long-term financial difficulties in the family or serious conflicts in the family increased the risk of work disability [6]. The abovementioned Swedish study found that sickness absence from school and childhood family receiving social benefits or being registered by the Child and Youth Welfare Committee were associated with sickness absence [8]. A UK study reported that cognitive ability, worrying about many things and recurrent abdominal pain at age 11 were associated with work disability whereas being miserable or having recurrent headaches were not [10]. A Finnish birth cohort study reported that parents’ psychiatric care or work disability due to mental disorders was associated with offspring long-term work disability due to mental disorders in early adulthood [11].

Previous studies have often addressed the question of whether adult socioeconomic position mediates between childhood factors and sickness absence. Both Norwegian and Swedish studies suggested that the contribution of childhood factors to sickness absence was partly mediated through own education [8,9] and socioeconomic position [8] but childhood factors seemed to also have direct effects. The Finnish study examining joint associations of childhood adversities and adult socioeconomic position found that childhood adversity and low adult socioeconomic position had an additive effect on work disability [6].

Previous research leaves some gaps regarding childhood factors and subsequent sickness absence. First, many studies have been conducted among rather young employees, neglecting older ones. Second, previous studies have not distinguished between self-certified and medically confirmed sickness absence. Medically-certified sickness absence is considered as a valid measure of health [12] whereas self-certified sickness absence might to a greater extent reflect other determinants of sickness absence such as working conditions [13] and health behaviours [14]. Third, the contribution of particular childhood adversities independently remains inadequately studied. Fourth, in addition to adult socioeconomic position the contribution of other temporally closer determinants of sickness absence such as working conditions and health behaviours and potential mechanisms behind the associations have not been examined.

The first aim of this study was to examine whether parental education and childhood adversities contribute to self-certified and medically confirmed sickness absence among midlife employees. The second aim was to examine whether adult socioeconomic position, marital status, working conditions and body mass index (BMI) and health behaviours contribute to the associations.

## Material and methods

This study is part of the Helsinki Health Study (HHS) on employees of the City of Helsinki [15], which with its 37,000 employees (76% women) is the largest employer in Finland. The jobs include both white- and blue-collar occupations in diverse fields of activities such as health care, social service, education, city planning and technical services. Data on parental education, childhood adversities and covariates were derived from the HHS baseline survey collected in 2000, 2001 and 2002 among employees who reached the age of 40, 45, 50, 55 or 60 years during those years. Altogether 8960 out of a targeted 13,346 employees participated and the response rate was thus 67%. The questionnaire data were linked with sickness absence data among respondents who gave their written consent (78%). There were 836 individuals who had retired, changed employer or died before the beginning of the follow-up of sickness absence and were thus excluded. After exclusions due to missing data on covariates (*n*=424) the data consisted of 5728 employees, of whom 4636 were women and 1092 were men. Item non-response concerning the questions on parental education and childhood adversities varied from 1% to 10% in the linked data and partially overlapped with other drop-out. The final number of participants in each analysis can be seen in Table I.

**Table I.** Characteristics of the data by parental education and childhood adversities.

	*n*	%	Person-years during follow-up	Number of sickness absence spells		Number of sickness absence spells/100 person years	
				Self-certified sickness absence	Medically-certified sickness absence	Self-certified sickness absence	Medically-certified sickness absence
**Paternal education**							
High	595	11	3000	3866	1543	129	51
Mid-level	1485	26	7823	11471	5466	147	70
Low	3554	63	18,158	25,664	14,416	141	79
**Maternal education**							
High	288	5	1461	1669	730	114	50
Mid-level	1241	22	6521	9337	4295	143	66
Low	4145	73	21,215	30,421	16,635	143	78
**Childhood economic difficulties**							
No	4352	82	22,493	30,515	15,646	136	70
Yes	929	18	4782	7900	4291	165	90
**Childhood illness**							
No	4846	93	25,095	34,858	17,950	139	72
Yes	385	7	1935	3285	1824	170	94
**Parental divorce**							
No	4630	89	23,949	33,013	17,041	138	71
Yes	597	11	3100	4949	2685	160	87
**Parental death**							
No	4570	88	23,742	33,313	17,205	140	72
Yes	648	12	3192	4530	2279	142	71
**Parental mental illness**							
No	4890	95	25,253	34,852	18,096	138	72
Yes	281	5	1504	2881	1377	192	92
**Parental alcohol problems**							
No	4234	80	21,731	29,430	15,223	135	70
Yes	1058	20	5615	9345	4793	166	85
**Bullying at school or by peers**							
No	4787	92	24,747	33,998	17,539	137	715
Yes	427	8	2220	4145	2148	187	97
**Adversity sum score**							
0	2971	53	15,368	19,984	10,322	130	67
1–3	2536	45	13,005	19,737	10,615	152	82
4+	122	2	629	1476	660	235	105
**Any adversity**							
No	2971	53	15,368	19,984	10,322	130	67
Yes	2658	47	13,634	21,213	11,275	156	83

According to the non-response analysis younger employees, employees in lower socioeconomic classes and employees with longer sickness absence during the study year were somewhat less likely to participate. Men in lower occupational classes and employees with medically-certified sickness absence were somewhat overrepresented among the non-consenters to data linkage. The differences were small and not fully consistent [15].

The Ethics Committee of the Department of Public Health at the University of Helsinki and the health authorities at the City of Helsinki approved the study.

### Parental education and childhood adversities

Maternal and paternal education were inquired by questions with six response alternatives and divided into three classes: ‘low’ (elementary school or part of it or intermediate school), ‘mid-level’ (vocational school or matriculation or college-level training) and ‘high’ (polytechnic or university degree). In the analyses examining joint associations of parental education and adult occupational class maternal and paternal education were dichotomized by combining ‘mid-level’ and ‘high’ education. Childhood adversities before the age of 16 years were asked by six questions with response alternatives ‘yes’ and ‘no’. The questions inquired whether the respondent had had serious or long-term illness during childhood, whether parents divorced or one of the parents died, whether one of the parents had mental health problems, whether parental alcohol drinking caused problems at home, whether the family had significant economic difficulties and whether the respondent had been bullied at school or by peers. Similar questions on childhood adversities have been used in previous studies by us and others [6,7,16].

### Sickness absence

The data on sickness absence spells, their dates and duration were derived from the employer’s personnel register. The follow-up started from the beginning of 2003 and continued until the end of 2008 or when the employee quit working for the City of Helsinki. The average follow-up time was 5.1 years. Self-certified (1–3 days) and medically-certified (>3 days) sickness absence spells were analysed separately as the City of Helsinki requires a medical certificate of sickness absence spells exceeding three days. Medical certification means that the employee must provide the employer with a medical certificate including a diagnosis and assessment of work ability done by a medical professional.

### Covariates

Age and gender were included as covariates. Marital status was divided into three categories: ‘single’, ‘married or cohabiting’ and ‘divorced or widowed’. Occupational class consisted of four categories: ‘manual workers’, ‘routine non-manual workers’, ‘semi-professionals’ and ‘managers and professionals’. In the analyses examining joint associations of childhood factors and adult occupational class occupational class was dichotomized by combining ‘manual’ and ‘routine non-manual’ workers and ‘semi-professionals’ and ‘managers and professionals’. Mental and physical workload were inquired with single-item questions about how heavy or light the respondent considered the work to be. Smoking was classified into current smoking and non-smoking. BMI was calculated from self-reported data of weight and height (weight/height^2^). Leisure-time physical activity was measured using four questions from which metabolic equivalent tasks (METs) were calculated. Problem drinking was measured by the CAGE-scale (cutting down on alcohol, annoyed by criticism, guilty and in need of an eye-opener) [17]. BMI, MET hours per week and CAGE were included as continuous variables.

### Statistical methods

The rates of self-certified and medically-certified sickness absence spells per 100 person-years were calculated for parental education and childhood adversities. The associations between parental education and childhood adversities and sickness absence were analysed using Poisson regression analysis. Follow-up time was included as an offset variable to take into account different follow-up time between the participants. The models showed over-dispersion and thus a scale-parameter obtained by dividing the residual deviance by the degrees of freedom was added. The numbers of self-certified and medically-certified sickness absence spells during the follow-up were the outcome measures. The results are presented as rate ratios (RRs) and their 95% confidence intervals (95% CIs). First we fitted base models adjusted for age, gender and marital status. Then occupational class, next working conditions and finally BMI and health behaviours were added to the base models in turn, that is, removing the previous covariate before adding a new one.

Joint associations of childhood factors and occupational class were examined. The models were adjusted for age, gender and marital status. To examine the synergistic interactions the synergy indices (S) were calculated using the following equation: S = RR (low adult occupational class, low parental education − 1)/[(RR high adult occupational class, low parental education − 1) + (RR low adult occupational class, high parental education − 1)]. A synergy index above 1 suggests that the joint association is synergistic, a synergy index equal to 1 suggests an additive association and a synergy index below 1 an antagonistic association.

Interactions for gender were tested (*p*<0.1), and significant interactions concerned maternal education and self-certified sickness absence (*p*=0.0286), occupational class and self-certified sickness absence (*p*=0.0317), occupational class and medically certified sickness absence (0.0587) and the joint association of occupational class and maternal education and self-certified sickness absence (*p*=0.0184). These analyses were performed separately for men and women. Otherwise women and men were pooled.

## Results

Low education was the most common paternal and maternal education type (Table I). The prevalence of childhood adversities ranged between 5% and 20%. Childhood economic difficulties and parental alcohol problems were the most common childhood adversities. The amount of self-certified sickness absence spells was higher than that of medically-certified sickness absence spells.

Among women both mid-level (RR 1.25, 95% CI 1.06–1.48) and low (1.33, 1.13–1.55) maternal education were associated with an increased risk of self-certified sickness absence after adjustments for age and for marital status (Table II). Adjustment for occupational class attenuated these associations whereas those for working conditions and BMI and health behaviours had only minor contributions. Childhood economic difficulties, childhood illness, parental divorce, parental mental illness, parental alcohol problems and bullying at school or by peers were associated with self-certified sickness absence. The risk of self-certified sickness absence was 31% higher among those with parental mental illness and 33% higher among those who had experienced bullying compared with those without these childhood adversities. Adjustment for occupational class abolished the association between parental divorce and self-certified sickness absence, otherwise adjustments for covariates had only minimal contributions.

**Table II.** The associations between parental education, childhood adversities and self-certified sickness absence. Rate ratios and their 95% confidence intervals.

	Age, gender, marital status = base model	Base model + occupational class	Base model + working conditions	Base model + BMI and health behaviours^a^
**Paternal education**				
High	1.00	1.00	1.00	1.00
Mid-level	1.09 (0.98–1.21)	1.01 (0.91–1.12)	1.07 (0.97–1.19)	1.09 (0.98–1.20)
Low	1.07 (0.97–1.17)	0.94 (0.85–1.03)	1.03 (0.94–1.13)	1.05 (0.96–1.15)
**Maternal education**				
**Women**				
High	1.00	1.00	1.00	1.00
Mid-level	1.25 (1.06–1.48)	1.17 (0.99–1.38)	1.22 (1.04–1.44)	1.25 (1.07–1.47)
Low	1.32 (1.13–1.55)	1.17 (1.00–1.37)	1.28 (1.09–1.49)	1.31 (1.12–1.52)
**Men**				
High	1.00	1.00	1.00	1.00
Mid-level	1.00 (0.72–1.38)	0.79 (0.59–1.06)	0.99 (0.72–1.38)	0.99 (0.73–1.35)
Low	0.89 (0.66–1.20)	0.93 (0.68–1.28)	0.89 (0.66–1.20)	0.88 (0.66–1.17)
**Childhood economic difficulties**				
No	1.00	1.00	1.00	1.00
Yes	1.22 (1.14–1.31)	1.19 (1.11–1.27)	1.20 (1.12–1.29)	1.17 (1.09–1.25)
**Childhood illness**				
No	1.00	1.00	1.00	1.00
Yes	1.23 (1.11–1.36)	1.21 (1.10–1.34)	1.22 (1.10–1.35)	1.19 (1.08–1.31)
**Parental divorce**				
No	1.00	1.00	1.00	1.00
Yes	1.11 (1.02–1.20)	1.06 (0.98–1.16)	1.10 (1.01–1.19)	1.07 (0.99–1.17)
**Parental death**				
No	1.00	1.00	1.00	1.00
Yes	1.05 (0.96–1.14)	1.02 (0.94–1.11)	1.03 (0.95–1.13)	1.02 (0.94–1.11)
**Parental mental illness**				
No	1.00	1.00	1.00	1.00
Yes	1.31 (1.18–1.46)	1.30 (1.17–1.44)	1.31 (1.18–1.46)	1.26 (1.14–1.40)
**Parental alcohol problems**				
No	1.00	1.00	1.00	1.00
Yes	1.17 (1.10–1.25)	1.15 (1.08–1.23)	1.16 (1.09–1.24)	1.13 (1.06–1.21)
**Bullying at school or by peers**				
No	1.00	1.00	1.00	1.00
Yes	1.33 (1.22–1.46)	1.30 (1.19–1.43)	1.32 (1.21–1.45)	1.28 (1.17–1.40)

a Health behaviours included smoking, problem drinking and leisure-time physical activity.

BMI: body mass index

Mid-level (1.32, 1.16–1.50) and low (1.48, 1.32–1.67) paternal education were associated with an increased risk of medically-certified sickness absence compared with high paternal education (Table III). Mid-level (1.26, 1.05–1.50) and low (1.49, 1.26–1.77) maternal education were associated with medically-certified sickness absence. Adjustment for occupational class abolished and adjustment for working conditions attenuated these associations whereas BMI and health behaviours had only minor contribution. Six of seven studied childhood adversities, namely childhood economic difficulties, childhood illness, parental divorce, parental mental illness, parental alcohol problems and having been bullied at school or by peers, were associated with an increased risk of medically-certified sickness absence. The risk of medically-certified sickness absence was 32% higher among those with childhood illness and 37% higher among employees who had experienced bullying compared with employees without these childhood adversities. Adjustment for occupational class slightly attenuated the associations, whereas further adjustments had only minimal contributions.

**Table III.** The associations between parental education, childhood adversities and medically-certified sickness absence. Rate ratios and their 95% confidence intervals.

	Base model = age, gender, marital status	Base model + occupational class	Base model + working conditions	Base model + BMI and health behaviours^a^
**Paternal education**				
High	1.00	1.00	1.00	1.00
Mid-level	1.32 (1.16–1.50)	1.09 (0.96–1.23)	1.25 (1.10–1.41)	1.27 (1.12–1.44)
Low	1.48 (1.32–1.67)	1.07 (0.95–1.20)	1.32 (1.18–1.49)	1.40 (1.24–1.57)
**Maternal education**				
High	1.00	1.00	1.00	1.00
Mid-level	1.26 (1.05–1.50)	1.05 (0.89–1.24)	1.16 (0.98–1.38)	1.24 (1.04–1.47)
Low	1.49 (1.26–1.77)	1.08 (0.92–1.27)	1.32 (1.12–1.56)	1.43 (1.22–1.68)
**Childhood economic difficulties**				
No	1.00	1.00	1.00	1.00
Yes	1.26 (1.17–1.36)	1.15 (1.07–1.24)	1.21 (1.12–1.30)	1.20 (1.11–1.29)
**Childhood illness**				
No	1.00	1.00	1.00	1.00
Yes	1.32 (1.18–1.47)	1.26 (1.14–1.40)	1.31 (1.17–1.45)	1.29 (1.16–1.43)
**Parental divorce**				
No	1.00	1.00	1.00	1.00
Yes	1.20 (1.09–1.32)	1.08 (0.99–1.18)	1.17 (1.07–1.28)	1.15 (1.06–1.26)
**Parental death**				
No	1.00	1.00	1.00	1.00
Yes	0.99 (0.89–1.09)	0.92 (0.84–1.01)	0.94 (0.85–1.03)	0.95 (0.86–1.05)
**Parental mental illness**				
No	1.00	1.00	1.00	1.00
Yes	1.22 (1.08–1.39)	1.22 (1.09–1.37)	1.22 (1.08–1.37)	1.19 (1.06–1.35)
**Parental alcohol problems**				
No	1.00	1.00	1.00	1.00
Yes	1.20 (1.11–1.29)	1.14 (1.06–1.22)	1.16 (1.08–1.25)	1.16 (1.08–1.25)
**Bullying at school or by peers**				
No	1.00	1.00	1.00	1.00
Yes	1.37 (1.24–1.52)	1.26 (1.14–1.39)	1.34 (1.21–1.48)	1.30 (1.18–1.44)

a Health behaviours included smoking, problem drinking and leisure-time physical activity.

BMI: body mass index

Having experienced any childhood adversity increased the risk of both self-certified (1.18, 1.12–1.25) and medically-certified (1.22, 1.15–1.30) sickness absence (Table IV). The associations remained after adjustments for covariates. The risk of both self-certified and medically-certified sickness absence increased with multiple childhood adversities. The risk of self-certified sickness absence was 67% and that of medically-certified sickness absence was 50% higher than among those with no childhood adversity. Adjustments for occupational class, working conditions and BMI and health behaviours somewhat attenuated the associations.

**Table IV.** The associations between any and multiple childhood adversities with self-certified and medically-certified sickness absence. Rate ratios and their 95% confidence intervals.

	Base model = age, gender, marital status	Base model + occupational class	Base model + working conditions	Base model + BMI and health behaviours^a^
**Self-certified sickness absence**				
Any childhood adversity *vs*. none	1.18 (1.12–1.25)	1.15 (1.09–1.22)	1.17 (1.11–1.23)	1.14 (1.08–1.20)
**Childhood adversity sum score**				
0	1.00	1.00	1.00	1.00
1–3	1.16 (1.09–1.22)	1.13 (1.07–1.19)	1.15 (1.08–1.21)	1.12 (1.06–1.18)
4+	1.67 (1.44–1.94)	1.60 (1.38–1.85)	1.65 (1.43–1.92)	1.53 (1.32–1.77)
**Medically-certified sickness absence**				
Any childhood adversity *vs*. none	1.22 (1.15–1.30)	1.13 (1.07–1.20)	1.18 (1.11–1.25)	1.17 (1.10–1.24)
**Childhood adversity sum score**				
0	1.00	1.00	1.00	1.00
1–3	1.21 (1.13–1.28)	1.12 (1.06–1.19)	1.17 (1.10–1.24)	1.16 (1.09–1.23)
4+	1.50 (1.25–1.80)	1.30 (1.10–1.54)	1.45 (1.21–1.72)	1.30 (1.09–1.55)

a Health behaviours included smoking, problem drinking and leisure-time physical activity.

BMI: body mass index

Low adult occupational class was associated with self-certified and medically certified sickness absence among both women (1.29, 1.22–1.37 for self-certified sickness absence and 2.00, 1.88–2.14 for medically-certified sickness absence) and men (1.54, 1.30–1.81 for self-certified sickness absence and 2.34, 1.98–2.77 for medically-certified sickness absence) (Table V). Adult occupational class dominated the joint associations. Low parental education only was not associated with sickness absence whereas childhood adversity increased both self-certified (1.21, 1.11–1.31) and medically-certified (1.14, 1.04–1.26) sickness absence without low adult occupational class. The joint associations of parental education/childhood adversity and adult occupational class with medically-certified sickness absence were synergistic.

**Table V.** The joint associations between parental education/childhood adversity and adult occupational class and self-certified and medically-certified sickness absence.

	*n*	%	Self-certified sickness absence		Medically-certified sickness absence	
			Number of sickness absences/100 person-years	RR, 95% CI Adjusted for age, gender and marital status	Number of sickness absences/100 person-years	RR, 95% CI Adjusted for age, gender and marital status
**Adult occupational class**						
**Women**						
High	2224	48	134	1.00	52	1.00
Low	2412	52	172	1.29 (1.22–1.37)	106	2.00 (1.88–2.14)
**Men**						
High	795	73	76	1.00	36	1.00
Low	297	27	127	1.54 (1.30–1.81)	88	2.34 (1.98–2.77)
**Maternal education**				0.0184		
Neither	1056	19			48	1.00
Low adult occupational class only	473	8			95	1.89 (1.68–2.13)
Low maternal education only	1941	34			48	1.01 (0.91–1.12)
Both	2204	39			105	2.08 (1.90–2.28)
Synergy index						1.20
**Women**						
Neither	798	17	132	1.00		
Low adult occupational class only	416	9	177	1.32 (1.18–1.48)		
Low maternal education only	1410	31	135	1.05 (0.96–1.15)		
Both	1968	43	171	1.34 (1.23–1.45)		
Synergy index				0.91		
**Men**						
Neither	258	24	84	1.00		
Low adult occupational class only	57	5	166	1.77 (1.31–2.39)		
Low maternal education only	531	49	72	0.89 (0.72–1.09)		
Both	236	22	117	1.32 (1.06–1.65)		
Synergy index				0.48		
**Paternal education**						
Neither	1428	25	122	1.00	49	1.00
Low adult occupational class only	652	21	185	1.40 (1.28–1.53)	99	1.91 (1.73–2.11)
Low paternal education only	1564	28	116	0.97 (0.90–1.05)	47	0.97 (0.88–1.07)
Both	1990	35	161	1.26 (1.17–1.35)	105	2.02 (1.86–2.20)
Synergy index				0.70		1.16
**Childhood adversity**						
Neither	1707	30	109	1.00	45	1.00
Low adult occupational class only	1264	22	158	1.35 (1.25–1.46)	97	2.01 (1.85–2.20)
Childhood adversity only	1260	22	133	1.21 (1.11–1.31)	52	1.14 (1.04–1.26)
Both	1398	25	176	1.50 (1.38–1.62)	110	2.31 (2.12–.2.50)
Synergy index				0.89		1.14

RR: rate ratio; CI: confidence interval

## Discussion

This study sought to examine the associations between parental education, childhood adversities and self-certified and medically-certified sickness absence among midlife municipal employees.

Both paternal and maternal low education increased the risk of medically-certified sickness absence whereas only maternal low education among women increased that of self-certified sickness absence. Our results are in line with previous studies [8–10], although previous studies have not examined paternal and maternal socioeconomic position separately but focused on paternal socioeconomic position [8–10] or a combined [8] measure. The reasons for the gender difference remain unclear but according to previous evidence self-certified sickness absence is more common among women [18] and low education is a known determinant of sickness absence [19]. Gender-segregated occupations might explain the gender difference to some degree, for example the study population included a lot of women doing physically demanding work in nursing and child-care. However, different occupation classes all included both women and men. Having experienced any and six of seven studied individual childhood adversities were associated with sickness absence. In line with a previous evidence no single adversity was outstanding [6]. Bullying at school or by peers showed strong associations with both self-certified and medically-certified sickness absence. Bullying has been previously associated with adult mental health problems [20], substance use [20] and pain [16]. Our study encourages further research on the longstanding health effects of bullying. Parental mental health problems were consistently associated with self-certified and medically-certified sickness absence. Parental mental health problems have been previously associated with offspring mental health problems [21]. Regarding work disability, a Finnish study found that parental mental health problems were associated with work disability in early adulthood. The association was mediated through mental disorder and social disadvantage in adolescence [11]. Offspring predisposition might play a role in addition to environment during growing up [22].

We examined both self-certified and medically-certified sickness absence as their determinants partly differ. In addition to health various factors at work and outside work such as motivational factors [23], working conditions [13] and work–family characteristics [24] are associated with them. Medically-certified sickness absence is more bound to health than self-certified sickness absence, the need for which is considered by the employee him/herself. In the present study parental education showed stronger associations with medically-certified sickness absence but otherwise the associations were rather similar. To our notice no theory behind the associations of childhood adversities and parental education with sickness absence exists. Childhood adversities such as childhood illness could have direct effects on adult health and thus lead to sickness absence. In addition, childhood factors might affect adult health indirectly by contributing to known determinants of adult health such as health behaviours and occupational class. In addition to health status various other factors such as working conditions [10] and motivational factors [24] contribute to sickness absence and childhood factors might act through these factors. In order to examine the pathways behind the associations we adjusted for known proximal determinants of sickness absence, namely occupational class [19], working conditions [13], BMI [14,25], health behaviours [14] and marital status [26]. Marital status was included as a potential mediator between childhood and sickness absence but it had essentially no contribution to the associations and was included in the base model. Occupational class mainly abolished the association between parental education and sickness absence and attenuated the associations between childhood adversities and medically-certified sickness absence, suggesting that the adult situation transmitted the association. This finding is in line with previous studies [8–10]. In line with a previous Finnish study [6] joint associations of parental education/childhood adversity and adult occupational class suggested that low parental education/childhood adversity and low adult socioeconomic class had a synergistic association with medically-certified sickness absence and thus accumulation of risk factors contributes to ill health in midlife. Otherwise the contribution of the covariates was minor. BMI and health behaviours slightly attenuated the associations and thus might have mediated the association between childhood factors and sickness absence. Working conditions were included as potential mediators and they had virtually no contribution to the associations between childhood adversities and sickness absence but slightly attenuated the association between parental education and sickness absence. The associations between childhood adversities and sickness absence remained after the adjustments and childhood adversity was associated with sickness absence even without low adult occupational class. The results thus suggest that childhood adversities might have direct long-term influence on sickness absence even decades later.

The strengths of the study include a large data set, register-based data on sickness absence, the possibility to differentiate between self-certified and medically-certified sickness absence spells, prospectively followed disability sickness absence after reporting parental education and childhood adversities, and the fact that we were able to control for several covariates. The major limitation was the retrospective and self-reported data of parental education and childhood adversities. Clear-cut questions such as parental divorce and death might be less biased whereas questions such as being bullied might be affected by mood and other conditions during the time of the survey. A review on adult recall of childhood adversities reported that false negative reports are common [27]. The fact that study participants were already in midlife and employed might mean that the associations are underestimated if people with more childhood adversities had retired or otherwise selected out of the workforce before midlife. The special interest of the present paper was, however, in midlife employees instead of the young.

In conclusion, this study showed that low parental education and childhood adversities contribute to both self-certified and medically-certified sickness absence still in midlife. Previous studies have shown that there are effective ways to promote well-being of families with children. For example, anti-bullying programmes effectively reduce school bullying [28], high-quality early childhood education benefits children’s development whereas low-quality childcare can produce a risk [29] and work-places with a family-supportive organizational culture are associated with lower levels of work–family conflict [30]. Paying attention to these and other measures promoting well-being of families with children might improve work ability and prevent sickness absence decades after childhood.
